# Strengthening Strategies to Recruit Racial/Ethnic Minority Populations for Health Research Studies

**DOI:** 10.1093/geroni/igab046.2394

**Published:** 2021-12-17

**Authors:** Roy Thompson, Thomas Konrad, Hanzhang Xu

**Affiliations:** 1 Duke University, Durham, North Carolina, United States; 2 University of North Carolina at Chapel Hill, Chapel Hill, North Carolina, United States; 3 Duke University School of Medicine, Duke University, North Carolina, United States

## Abstract

With the current spotlight on systemic racism and the need to address health inequities, it is critical to develop culturally appropriate strategies for recruiting research study participants from racial/ethnic minority groups. Empirical studies have highlighted that people from racial/ethnic minority groups have poorer health outcomes compared to non-Hispanic Caucasians. However, racial/ethnic minority groups remain underrepresented in healthcare research. Several factors may contribute to the lower participation of racial/ethnic minority groups. Sequelae of atrocities in healthcare research on African American/Black people in the US during slavery and Jim Crow eras were widespread and persistent. Discrimination against people of Hispanic descent and increased anti-Asian discrimination have also been documented. Fear and mistrust of the health system and researchers have been identified as critical barriers to participation in clinical research for these populations. Further, health research teams rarely reflect the racial/ethnic diversity of the US population, hindering diversity in recruiting study participants. Inadequate ethnic/racial minority groups participation in study populations not only weakens external validity of empirical studies, but research interventions and policies being implemented may not be culturally appropriate to all populations. Therefore, systemic strategies to improve recruitment of racial/ethnic minority groups should: 1) increase preferential funding to incentivize research teams becoming more racially/ethnically diverse; 2) increase recruitment of racial/ethnically diverse healthcare researchers; 3) use community-based participatory research designs to build trust among racial/ethnic minority populations; 4) provide training on culturally appropriate research study recruitment strategies to the academic communities; 5) apply a prism of intersectionality for representation throughout the research cycle.

